# Emotional problems among recent immigrants and parenting status: Findings from a national longitudinal study of immigrants in Canada

**DOI:** 10.1371/journal.pone.0175023

**Published:** 2017-04-04

**Authors:** Dillon T. Browne, Aarti Kumar, Sofia Puente-Duran, Katholiki Georgiades, George Leckie, Jennifer Jenkins

**Affiliations:** 1 Department of Applied Psychology and Human Development, University of Toronto, Toronto, Ontario, Canada; 2 Department of Psychology, Ryerson University, Toronto, Ontario, Canada; 3 Faculty of Health Sciences, McMaster University, Hamilton, Ontario, Canada; 4 Center for Multilevel Modeling, University of Bristol, Senate House, Tyndall Avenue, Bristol, United Kingdom; Public Health Agency of Canada, CANADA

## Abstract

The present study examined predictors of emotional problems amongst a nationally representative cohort of recent immigrants in Canada. Specifically, the effects of parenting status were examined given the association between parenting stress and mental health. Data came from the Longitudinal Survey of Immigrants to Canada (N = 7055). Participants were recruited 6-months post landing (2001–2002) and followed up at 2 and 4 years. Self-reported emotional problems over time were considered as a function of parenting status (Two Parent, Lone Parent, Divorced Non-Parent, Non-Divorced Non-Parent) and sociodemographic characteristics. Odds of emotional problems were higher among Two Parent, OR = 1.12 (1.01, 1.24), Lone Parent, OR = 2.24 (1.75, 2.88), and Divorced Non-Parent, OR = 1.30 (1.01, 1.66) immigrants compared to Non-Divorced Non-Parents. Visible minority status, female gender, low income, and refugee status were associated with elevated risk. Findings reveal that immigrant parents are at risk for emotional health problems during the post-migration period. Such challenges may be compounded by other sociodemographic risk.

## Introduction

There is an ongoing need for mental health research that keeps pace with changes in global immigration patterns, particularly among immigrants who are parents [1]. Migration stress is linked to depressive symptoms in parents [2, 3]. However, no studies have examined if immigrant parents experience *more* emotional problems in comparison to those without children. The stressors associated with parenting are well documented [4, 5] and linked to environmental risk factors, such as socioeconomic disadvantage, and to mental health problems, such as depression [6]. However, much of this evidence is based on non-immigrant populations. Immigrant parents may be at elevated risk, given that they are not only dealing with parenting stressors, but also the stressors of migration and resettlement. Furthermore, there may be unique pre-migration stressors that place immigrants at risk (e.g. political upheaval, exposure to violence, poverty). Such challenges may be exacerbated when parents are responsible for raising children. Thus, the objective of this study is to compare the likelihood of emotional problems among recent immigrant parents versus non-parents, while simultaneously considering sociodemographic factors that may impact mental health.

Findings from longitudinal studies of immigrant mental health have been equivocal. Different investigations have reported increasing, decreasing, and stable prevalence rates following resettlement [7–13]. These divergent findings may be attributable to heterogeneity of immigrant samples. Specifically, there may be differential patterns of post migration emotional problems as a function of immigrant classifications (e.g. refugee, economic, family), sociodemographic characteristics (e.g. ethnicity, income, education), host country (e.g. Canada, USA, Europe), and other structural characteristics, such as caring for dependents (i.e. parents versus non-parents).

Parents tend to exhibit higher levels of mental health problems than non-parents [6, 13]. Parenting stressors are more likely to occur in settings of risk (e.g. poverty) and during periods of life transition and struggle [4, 5]. Given the challenges of immigration and resettlement, immigrant parents likely experience greater stress and emotional problems compared to non-parent immigrants. Caregiving stressors may also build upon the challenges parents faced in their native countries. While both mothers and fathers are at a heightened risk for depression in response to caregiving stress [14, 15], immigrant mothers are at particular risk due to post-natal mood fluctuation [16]. Additionally, immigrant women are more likely to care for children compared to men, resulting in fewer opportunities for integration, exposure to new environments, and employment [17]. Furthermore, they may be more subject to cultural expectations such as personal sacrifice and family service.

A number of other factors must be considered when conceptualizing the mental health of immigrants. For example, single relationship status is associated with depression amongst immigrants [18]. This may be exacerbated when singlehood involves children or is due to divorce, but may be associated with lower levels of risk when singlehood is continuous or does not involve children [19]. Currently, there is a dearth of literature studying single parenthood amongst immigrants. Also, while all immigrants share common stressors, visible minorities have greater difficulty adapting to host societies and report greater feelings of discomfort [20]. They also report greater discrimination [21–23] and lower self-esteem [24]. Notably, financial hardship has been identified as one of the strongest determinants of emotional problems [25, 26]. Studies find that North American immigrants are often materially disadvantaged [2, 27], a pattern that is exacerbated when immigrants are visible minorities and recently arrived [28, 29]. Furthermore, the expected link between income and education is not seen among immigrants. Schellenberg [30] found that new Canadian immigrants had unexpectedly low incomes despite high education. Many immigrants in North America have difficulty finding jobs that match their educational attainment [30, 31]. These challenges must be considered in concert with pre-migratory stressors. That is, asylum seekers may present to host nations with particular mental health challenges (e.g. post traumatic stress) due to adversity in their native countries [18].

### The present study

This prospective study examines emotional problems amongst recent immigrants to Canada as a function of parenting status. It was hypothesized that immigrant parents (especially those who are single) would have the highest odds of experiencing emotional problems compared to non-parents. This hypothesis was expected to operate over and above sociodemographic predictors of emotional problems (female gender, visible minority, low income and refugee). Based on data availability, trajectories of emotional problems from 6 months to 4 years post arrival are examined.

## Materials and methods

### Sample

The present sample was derived from Statistics Canada’s Longitudinal Survey of Immigrants to Canada (LSIC), which examines adjustment patterns among recent immigrants [32]. Participants were identified from the Citizenship and Immigration Canada database of all landed immigrants to Canada. The sample was created using a stratified sampling method and followed 7716 respondents across 3 time points (6 months, 2 years, and 4 years post-landing). Eligibility criteria included landing between October 1^st^, 2000 and September 20^th^, 2001 from abroad. Statistics Canada, operating under the Statistics Act [33], was responsible for ethical approval of the entire LSIC, in addition to conducting the survey and managing/storing data. Ethical approval for this study (a secondary analysis) was granted by the University of Toronto Research Data Center. Immigrants provided written informed consent at immigration, permitting Statistics Canada to contact them via telephone.

Interviews took place at 6 months (April 2001 to May 2002), 2 years (December 2002 to December 2003) and 4 years (November 2004 to November 2005) post-landing, respectively. Data were collected in person or by telephone using computer-assisted interviewing in 1 of 15 possible languages. Interviews lasted 1 to 1.5 hours. The average age of study participants was 34.93 years (SD = 11.34) at 6-months post immigration. Consistent with PLOS One’s data sharing policy, data are publically available from Canadian Research Data Center Network (CRDCN; http://www.statcan.gc.ca/eng/rdc/data; https://crdcn.org/contact-us)with the same permissions that were granted authors (see S1 Appendix for permissions and Acknowledgments section for full contact info).

### Measures

#### Emotional problems

Respondents were asked the following questions at 6 months, 2 years and 4 years, respectively: “Since you came to Canada, have you had any emotional or mental problems?” “Since your last interview, have you experienced any emotional problems? By emotional problems, I mean persistent feelings of sadness, depression, loneliness, etc.” “In the past 12 months, have you experienced any emotional problems? By emotional problems, I mean persistent feelings of sadness, depression, loneliness, etc.” The response was binary (Yes/No).

#### Parenting status

Parenting status fell into 4 categories: *Two parent*, *Lone parent*, *Divorced non-parent*, and *Non-divorced non-parent*. *Two parent* was defined as caring for one or more children (i.e. person equal to or less than 18 years of age) at any time point and identifying themselves as either “married” or “common-law” at all time points or “single” to “married” at any time point. *Lone parent* was defined as caring for one or more children at any time point and identifying themselves as “divorced, separated or widowed” at any time point. *Divorced non-parent* was defined as not caring for children at all time points and being “divorced, separated or widowed” at any time point. The reference category is respondents who are not caring for children and are either partnered or never married at all time points (*Non-divorced Non-parent*). Parenting status variables were mutually exclusive (see S2 Appendix for further description).

#### Covariates

*Time* was assessed with the following values: 0 (6-months post-arrival), 1 (2-years post-arrival), and 2 (4-years post-arrival). *Age* of respondents was assessed at 6-months. *Female gender* was represented by a dummy variable. Monthly *income* was assessed in Canadian dollars. *Education* was measured in years of full-time education, inside and outside Canada, excluding kindergarten. Immigrants fell into five categories: (1) *Family Class*, including their spouses, fiancés, parents, grandparents, dependents, and other family, (2) *Business Immigrants*, including their spouses and dependents, (3) *Refugees*, (4) *Skilled Workers*, including their spouses and dependents, and (5) *Other Immigrants*. Dummy variables were created and *Skilled Workers* was the reference category. Ethnicity fell into 7 categories: (1) *White*, (2) *South Asian*, (3) *East Asian* (Chinese, Japanese, Southeast Asian and Korean), (4) *Black*, (5) *Arab/West Asian*, (6) *Latin American*, and (7) *Filipino*. Dummy variables were created and *White* immigrants were the reference category. See S2 Appendix for additional description of measures.

### Analytic plan

The aforementioned predictors (with the exception of income) were treated as time invariant. This was due to low rates of change for certain variables (e.g. parenting status). Emotional problems were modelled as a function of response variables using multilevel mixed effects logistic regression, which is the standard subject-specific approach for modeling outcomes that are both repeated measures and dichotomous [34–36]. Model building took place in three steps: (1) ethnicity, (2) other covariates, and (3) parenting status. Model fit was evaluated using the Likelihood Ratio Test, Akaike’s Information Criterion (AIC) and the Bayesian Information Criterion (BIC).

### Missing data

Of the original longitudinal sample (*N* = 7716), 86 respondents were removed due to identifying ethnicity as *multiple visible minorities*, *don’t know* or *refusal*. It was deemed inappropriate to report outcomes on this heterogeneous group. An additional 575 were removed for missing data on emotional problems or income at 6-months, 2 years or 4 years. Although the analysis permits the inclusion of participants with incomplete data, it is the policy of Statistics Canada to “prevent the publication or disclosure of any information deemed to be confidential” (www.statcan.gc.ca). This includes small-size phantom cells which may inadvertently result in residual identity disclosure. Thus, these respondents were removed from the analysis. The final sample consisted of 7055 longitudinal respondents, which represented 91.43% of the initial sample. See S2 Appendix for additional discussion of missing data analysis.

## Results

Weighted descriptive statistics for dichotomous variables are presented in Table 1. Results are presented in accordance with Statistics Canada’s guidelines to privacy. Accordingly, descriptive data were weighted, while data in models could be weighted or unweighted (see S2 Appendix for details). The overall prevalence of emotional problems (EP) was 5.17% at 6-months, rising to 30.26% and 28.77% at 2-years and 4-years, respectively.

**Table 1:** Weighted frequencies of key demographic variables in the sample.

Variable	N	Percent
Emotional Problems (6-months)	364	5.17
Emotional Problems (2-years)	2134	30.26
Emotional Problems (4-years)	2029	28.77
Ethnicity:		
White	1471	20.85
South Asian	1851	26.25
East Asian	1910	27.08
Black	333	4.73
Arab	775	10.99
Latin America	192	2.73
Filipino	519	7.37
Female	3538	50.16
Low Income (Time 1)	1485	21.05
Low Income (Time 2)	1226	17.38
Low Income (Time 3)	1235	17.51
Immigration Category:		
Skilled Worker	4381	62.11
Family Class Immigrant	1787	25.34
Business Immigrant	359	5.10
Refugee	440	6.25
Other Immigrant	85	1.21
Parenting Status:		
Non-Parent (partnered or never married)	2256	31.99
Two Parent	4315	61.17
Lone Parent	191	2.72
Divorced Non-Parent	290	4.11

Note. Percentages are calculated as a proportion of the total sample N = 7055. N’s roundest to nearest whole number, as results are presented in accordance with Statistics Canada’s guidelines to avoid breach of privacy. Accordingly, descriptive data were weighted.

A null model was fit including only an intercept in the fixed and random parts of the model. The likelihood ratio test comparing the multilevel model with a logistic regression was significant χ^2^ (1) = 400.77, *p* < .001, indicating that a model that accounted for within-subject data-dependency was necessary. In other words, persons with EP at one time are more likely to have EP at another time. This non-independence of serial observations across respondents highlights the importance of considering the longitudinal patterns of EP among cohorts and prospectively studying individual functioning over time.

**Fig 1:**
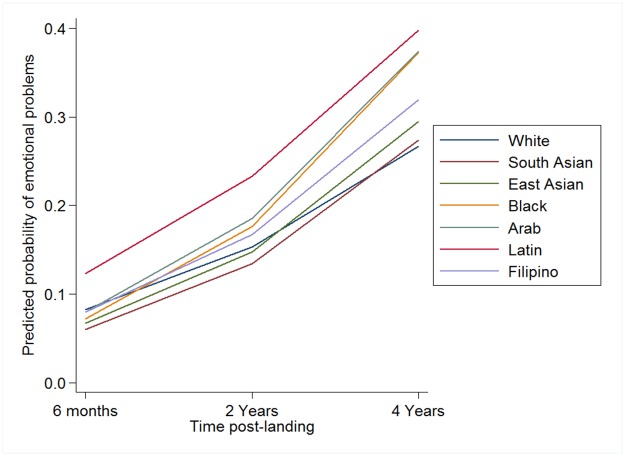
Predicted probability of reporting emotional problems from random-intercept logistic regression model as a function of ethnicity.

Model 1 examined how the overall relationship between the prevalence of EP and time varied by ethnic group. This model was a significant improvement over the null model based on the Likelihood Ratio Test, χ^2^ (14) = 33.16, *p* < .001, and a reduced AIC and BIC. The significant random intercept and intraclass correlation indicates that, after accounting for ethnic differences, 25% of the variability in EP is between-persons, while 75% is within-persons. Fig 1 presents the fitted probabilities from this model. The figure highlights several key patterns. First, the prevalence of EP increased over time for all ethnic groups. Second, Latin immigrants reported the highest prevalence of EP at each occasion. Third, Black and Arab immigrants showed the most rapid increase in EP over time, while White immigrants showed the least rapid increase. Table 2 presents the odds ratios and 95% CI for this model, allowing us to explore the significance (or not) of these patterns. At 6-months post immigration, the odds ratios show that relative to White immigrants (who have an odds of reporting EP of 0.09), only South Asians and East Asians were significantly less likely to report EP and only Latin immigrants were significantly more likely to report EP; Black, Arab and Filipino immigrants were no more or less likely than White immigrants to report EP 6-months post immigration. The odds ratios also show significantly more rapid increases in reporting EP over time among South Asian, East Asian, Black, and Arab immigrants compared to White immigrants; the increase in EP over time among Latin and Filipino immigrants was no more or less rapid than that for White immigrants.

**Table 2:** Maximum likelihood estimates for random-intercept logistic regression model for reporting emotional problems across the first four years in canada.

	Model 1		Model 2		Model 3	
	OR	(95% CI)	OR	(95% CI)	OR	(95% CI)
*Fixed Part* ^†^						
Ethnicity: ^a^						
South Asian	0.71**	(0.57, 0.89)	0.90	(0.71, 1.13)	0.91	(0.73, 1.15)
East Asian	0.80*	(0.65, 1.00)	0.89	(0.71, 1.11)	0.90	(0.72, 1.12)
Black	0.86	(0.61, 1.21)	0.77	(0.54, 1.10)	0.73	(0.51, 1.04)
Arab	0.96	(0.75, 1.23)	0.83	(0.64, 1.08)	0.85	(0.65, 1.10)
Latin	1.56*	(1.04, 2.35)	1.42	(0.95, 2.13)	1.40	(0.93, 2.10)
Filipino	0.96	(0.70, 1.33)	1.18	(0.84, 1.65)	1.20	(0.86, 1.68)
Time (Study Wave)	2.01***	(1.83, 2.22)	1.83***	(1.62, 2.07)	1.84***	(1.62, 2.08)
Ethnicity*Time: ^b^						
South Asian*Time	1.21**	(1.05, 1.39)	1.20*	(1.03, 1.39)	1.20*	(1.03, 1.39)
East Asian*Time	1.20*	(1.04, 1.37)	1.15	(1.00, 1.33)	1.16*	(1.00, 1.33)
Black*Time	1.38**	(1.11, 1.72)	1.36**	(1.09, 1.71)	1.37**	(1.10, 1.72)
Arab*Time	1.31**	(1.12, 1.54)	1.30**	(1.10, 1.54)	1.30**	(1.10, 1.54)
Latin*Time	1.08	(0.83, 1.41)	1.07	(0.82, 1.40)	1.07	(0.82, 1.40)
Filipino*Time	1.16	(0.95, 1.43)	1.13	(0.91, 1.40)	1.13	(0.91, 1.40)
Age			1.00	(0.99, 1.01)	1.00	(0.99, 1.01)
Female			1.47***	(1.26, 1.72)	1.43***	(1.22, 1.65)
Low Income			1.18	(0.98, 1.42)	1.18	(0.98, 1.42)
Education			1.02	(1.00, 1.05)	1.02	(1.00, 1.05)
Immigration category: ^c^						
Family Class			0.66***	(0.53, 0.81)	0.67***	(0.54, 0.83)
Business			0.62**	(0.43, 0.90)	0.63*	(0.44, 0.91)
Refugee			1.74***	(1.35, 2.23)	1.70***	(1.33, 2.19)
Other			1.05`	(0.50, 2.21)	1.06	(0.50, 2.12)
Age*Time			1.00	(1.00, 1.00)	1.00	(1.00, 1.00)
Female*Time			1.12*	(1.02, 1.24)	1.13*	(1.02, 1.24)
Low Income*Time			1.16*	(1.02, 1.32)	1.14*	(1.01, 1.30)
Education*Time			1.00	(0.99, 1.02)	1.00	(0.99, 1.02)
Immigration category*Time: ^d^						
Family Class*Time			1.08	(0.94, 1.24)	1.08	(0.94, 1.24)
Business Class*Time			1.13	(0.90, 1.43)	1.13	(0.90, 1.43)
Refugee*Time			0.92	(0.78, 1.08)	0.92	(0.78, 1.09)
Other Immigra*Time			1.10	(0.68, 1.78)	1.10	(0.68, 1.77)
Parenting status: ^e^						
Two Parent					1.12*	(1.01, 1.24)
Lone Parent					2.24***	(1.75, 2.88)
Divorced Non-Parent					1.30*	(1.01, 1.66)
	Est.	SE	Est.	SE	Est.	SE
*Random Part*						
Random Intercept	1.10***	(0.04)	1.03***	(0.04)	1.02***	(0.04)
Intraclass correlation	0.25		0.24		0.24	
*Model Fit*						
Log likelihood	-10233.64		-9974.63		-9954.13	
*Df*	15		31		34	
AIC	20497.28		20011.26		19976.26	
BIC	20616.68		20257.58		20246.42	

* *p*< .05,

**p < .01,

***p < .001

^†^ The estimate of the exponentiated constant is 0.09 (0.08, 0.10) in model 1, 0.07, (0.06, 0.09) in model 2 and 0.06, (0.05, 0.08) in model 3.

^a^ Reference category white

^b^ Reference category White*Time

^c^ Reference category Skilled worker

^d^ Reference category Skilled Worker*Time

^e^ Reference category Non-Divorced Non-Parents

*Note*. OR = Odds Ratio

Model 2 examined the impact of demographic covariates. This model was a significant improvement over the previous model based on the likelihood ratio test, χ^2^ (16) = 259.01, *p* < .001, in addition to lower values of the AIC and BIC. The odds of reporting EP did not differ as a function of age initially or over time. At 6-months post-migration, females had higher odds of reporting EP than male immigrants. Over time, the odds of reporting EP increased at a higher rate for female immigrants in comparison to men. The odds of reporting EP were the same for low and high-income immigrants initially. However, low-income respondents’ odds of reporting EP increase significantly more from cycle to cycle, relative to non-low-income respondents. Respondent’s level of education was not found to impact EP initially or over time. Refugees’ odds of reporting EP at 6-months post-arrival is significantly higher in comparison to skilled workers. On the other hand, family class immigrants and business class reported odds of EP that were lower in comparison to Skilled Workers. Although we examined whether being in different immigrant categories was associated with a change in prevalence over time (the interaction of immigrant class and time) no evidence for this was found.

**Fig 2:**
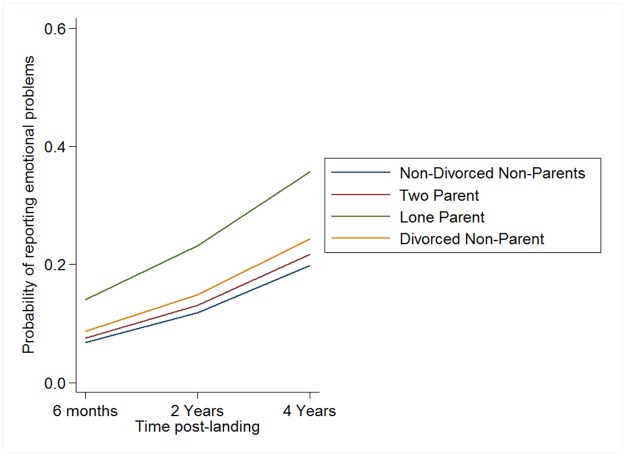
Predicted probability of reporting emotional problems from random-intercept logistic regression model as a function of parenting status.

Finally, in model 3 the impact of the parenting variables was examined. Two parent respondents had higher odds of reporting EP in comparison to respondents that were non-divorced non-parents. Lone parent respondents had odds of reporting EP that were over twice as high in comparison to respondents that were non-divorced, non-parents. Finally, Divorced, non-parents had odds of reporting emotional problems that were significantly higher than non-divorced non-parents (see Fig 2). Note that the confidence intervals for lone parents did not overlap with two parent or divorced non-parent respondents, indicating that this group has the highest odds of reporting EP. The interactions between parenting status and time were non-significant and removed due to compromised model fit. Thus, the final model was an improvement over Model 2 based on the likelihood ratio test, χ^2^ (3) = 20.5, *p* < .001, and a lower AIC and BIC. Note that sensitivity analyses were conducted in order to ensure the robustness of our findings to various model assumptions. These are described in the S2 Appendix.

## Discussion

The present study documents a substantial increase in emotional problems amongst a representative cohort of immigrants following arrival in Canada. By 2 years post-migration, nearly 1-in-3 immigrants in Canada is reporting emotional problems. Parents have even higher odds of reporting emotional problems compared to non-parents, and lone parents are at the greatest risk. These effects were evident after controlling for relevant socio-demographic characteristics (ethnicity, age, gender, socioeconomic status, and immigrant category), suggesting an effect of caregiver strain, and not simply an effect of financial burden or pre- and post-immigration hardship. Findings also reveal ethnic differences, where South Asians and East Asians initially have lower odds of reporting emotional problems compared to White immigrants, while Latin immigrants were higher. Over time, the likelihood of emotional problems increased more for visible minority immigrants (except Latin & Filipino) compared to White immigrants. Refugee status, female gender, and low income also contributed additional risk either initially or over time.

In the present study, greater emotional problems among parents corresponds to previous research linking parental stress and mental health problems among non-immigrants [6]. Also, it has been demonstrated that married persons report fewer mental health problems in comparison to their unmarried counterparts [37]. Furthermore, previous research suggests that unmarried persons with dependent children report more mental health problems than their married peers [38]. Collectively, these findings align with the current investigation, where single parents have the highest rates of emotional problems. Single parents’ distress appears to be due in large part to the fewer available social resources [18, 39]. It is worth noting that the observed differences in mental health across family structure were evident by 6-months amongst study families, highlighting the importance of supporting caregivers in the initial months post-landing.

Immigrants who reported being divorced with no children were also more likely to report emotional problems compared to Non-parents who were either partnered or never married. Divorced individuals report more emotional problems and stressors than married respondents, and some of this effect is attributable to pre-divorce adjustment [40]. Also, domestic violence has been identified as one of the reasons for divorce amongst immigrants, suggesting that multiple life stressors may explain the high rate of vulnerability in this group [41].

An important contribution of the current study was to examine emotional problems across ethnicities. Respondents who identified as South Asian and East Asian had lower odds of endorsing emotional problems at 6-months post arrival compared to White immigrants. Asian immigrants often arrive to receiving countries with many skills and strengths that helped them gain immigration candidacy [42], which corresponds to lower level of mental health problems [43]. Over time, however, South and East Asians, as well as Black, and Arab immigrants’ likelihood of reporting emotional problems increased more compared to White immigrants. This increased risk over time may be due to unmet expectations, in conjunction with specific obstacles, including, racism, discrimination, inequality, and unemployment [44].

Our findings revealed that rates of emotional problems systematically varied as a function of gender, income and immigration category. Compared to males, females were more likely to report emotional problems six months after immigration, and their odds of reporting emotional problems increased at a faster rate. Other research has documented a higher prevalence of internalizing psychopathology (depression and anxiety) in females during the period following immigration [45]. Furthermore, the rates of emotional problems increased most rapidly for those in lower socioeconomic strata. The effects of socioeconomic status on mental health are complex and multifaceted, operating through a variety of mechanisms including access to resources, health related behaviours, and psychosocial stress [46]. Socioeconomic disparities in numerous health outcomes have been documented in North America, where children and families living in lower socioeconomic strata report lower levels of health [27]. Though not evident at 6-months post-migration, the effect monthly income on the rate of change in emotional problems suggests income disparities emerge within 2 years of arrival. Additional research is needed that examines this trend in concert with pre-immigration socioeconomic status, paying particular attention to the social mobility patterns of single parent families, divorced immigrants and mothers.

Family Class Immigrants and Business Immigrants were less likely to report emotional problems in comparison to Skilled Workers. Perhaps Family Class Immigrants receive more social support from sponsoring family members? Previous research indicates that social support and community cohesion have a positive impact on mental health outcomes among immigrants [47]. For business immigrants, it is possible that self-employment coincides with greater economic capital and related resources that supports their success in settlement. Conversely, refugees had higher odds of emotional problems. This is consistent with research that reports greater emotional problems amongst refugees in comparison to other immigrants [9]. Although refugees constitute a minority Canadian immigrants, their relatively high mental health needs warrant close attention [18].

What can host countries do to best support immigrants who are also resettling with dependents? Our findings speak to the importance of aiding and assisting immigrants in their caregiving roles, especially when they are female, single, refugees, or of low income status. After decades of research, Beiser [18] has developed comprehensive policy recommendations, which include universal supports, in addition to the screening of immigrants for the purposes of providing psychosocial services based upon need. Resettlement is not a discrete event and immigrants’ needs change over time. For example, embeddedness in culturally similar social networks appears to be critical during the early stages of resettlement, while language acquisition opportunities foster subsequent social integration. Furthermore, given the centrality of economic challenge in caregiving stress, policy initiatives may also benefit from the inclusion of income supplements, child care benefits, and employment opportunities that are specifically targeted towards immigrant parents. Of course, the emotional health and wellbeing of all immigrants will be best supported in societies where our political representatives lead with the values inclusion, tolerance, and the embrace of cultural diversity. It is the responsibility of host nations to move policy initiatives beyond the adjudication of immigrant candidacy, and towards the provision of supports that foster the healthy resettlement of families and, ultimately, the success of societies.

### Limitations and future directions

There are several limitations of the current study. First, there is limited specificity of the outcome variable due to reliance on a single item. However, single item measurement is common in mental health epidemiology and previous studies have shown good convergence of these items with more detailed measurement [48]. Secondly, there may be some measurement bias due to the reliance on self-reports [49]. Nevertheless, these biases tend to be in the direction of under-reporting, suggesting that the obtained estimates are conservative [49]. Third, there are challenges with measuring depression across ethnicities. Some groups tend to express depressive symptoms as somatization, which could lead to under reporting. If items function differently across ethnicities, it is possible that ethnic differences are partly a function of measurement. Furthermore, results must be interpreted with caution as the wording of the outcome variable changed between 6-months and 2 years post-migration. It is possible that the increases in emotional problems from 6-months to 2 years are related to a better understanding of the question, or an increase in willingness to report emotional problems. Additional time points would have permitted us to further examine the shape of change in emotional problems beyond a linear model. Longitudinal research examining trajectories of emotional problems amongst immigrant parents beyond 3 waves of measurement is required. Fourth, parental status was used as a proxy for parenting stress. It is possible that the different categorizations of parenting may actually represent different mediating mechanisms (e.g. two-parent families having more marital discord, single-parent families representing parent-child dysfunction, etc.) The LSIC did not have measurement on all of these possible mechanisms. Future research is needed to clarify the *pathways* through which parenting status impacts mental health amongst immigrants. Finally, due to the nature of the LSIC, comparisons between sub-groups were limited. The dataset did not allow us to examine parents who were not immigrants, or evaluate the effects of ethnicity outside of the context of immigration. Similarly, there were a number of potential constructs of interest that were not measured in the LSIC, including additional information regarding household demographics (e.g. how many children at home), psychological adjustment of children, or whether or not there were children left in the home country. Future research should consider these additional modifying factors.

These limitations are balanced by the strengths of the LSIC, where measurement brevity is balanced by a large nationally representative sample that is followed for 4 years. Results are generalizable to the population of recent immigrants in Canada. Future epidemiological and clinical studies should attend to the effects of culture, values, beliefs, and attitudes surrounding acculturation. This will help shed light on why certain ethnicities are more vulnerable to emotional problems in Canada. Future research is needed to replicate findings showing that visible minority immigrants report higher odds of emotional problems over time compared to White immigrants. This is one of the few Canadian studies to observe ethnic disparities in mental health outcomes among immigrants. Finally, it is also important to understand the consequences of caregiver strain, as highly stressed parents endorse elevated levels of depression, anxiety and psychological distress, and tend to have children with the poorest developmental outcomes compared to parents who are less stressed [6, 50]. Overall, there is a pressing and continuous need for investigations on the mental health of very recent immigrants to Canada, Europe and the USA for parents and non-parents, alike, particularly in light of ongoing changes in global immigration policy.

## Supporting information

S1 Appendix Author permissions to access data from statistics canada. (PDF)

S2 Appendix Supplementary study information. (DOCX)

## References

[1] Yoshikawa H. Immigrants raising citizens: Undocumented parents and their children. New York: Russell Sage Foundation; 2011.

[2] Beiser M, Hou F, Hyman I, Tousignant M. Poverty, family process, and the mental health of immigrant children in Canada. American Journal of Public Health. 2002;92:220–7. 11818295 10.2105/ajph.92.2.220 PMC1447046

[3] Zelkowitz P, Schinazi J, Katofsky L, Saucier JF, Valenzuela M, Westreich R, et al Factors associated with depression in pregnant immigrant women. Transcultural psychiatry.2004;41(4):445–64. 10.1177/1363461504047929 15709645

[4] Abidin RR. Parenting Stress Index (PSI). Third Edition ed. Odessa, Fla: Psychological Assessment Resources, Inc; 1995.

[5] Newland RP, Crnic KA, Cox MJ, Mills-Koonce WR. The family model stress and maternal psychological symptoms: Mediated pathways from economic hardship to parenting. Journal of Family Psychology. 2013;27(1):96 10.1037/a0031112 23421837 PMC8011847

[6] Evenson RJ, Simon RW. Clarifying the Relationship Between Parenthood and Depression. Journal of health and Social Behavior. 2005;46(4):341–58. 10.1177/002214650504600403 16433280

[7] Breslau J, Chang DF. Psychiatric disorders among foreign-born and US-born Asian-Americans in a US national survey. Social Psychiatry and Psychiatric Epidemiology. 2006;41(12):943–50. 10.1007/s00127-006-0119-2 16988789 PMC2748988

[8] Kirmayer LJ, Narasiah L, Munoz M, Rashid M, Ryder AG, Guzder J, et al Common mental health problems in immigrants and refugees: general approach in primary care. Canadian Medical Association Journal. 2011;183(12):E959–E67. 10.1503/cmaj.090292 20603342 PMC3168672

[9] Pernice R, Brook J. Refugees' and immigrants' mental health: Association of demographic and post-immigration factors. The Journal of social psychology. 1996;136(4):511–9. 10.1080/00224545.1996.9714033 8855381

[10] Setia MS, Quesnel-Vallee A, Abrahamowicz M, Tousignant P, Lynch J. Different outcomes for different health measures in immigrants: evidence from a longitudinal analysis of the National Population Health Survey (1994–2006). Journal of immigrant and minority health. 2012;14(1):156–65. 10.1007/s10903-010-9408-7 21042935

[11] Tiwari SK, Wang J. Ethnic differences in mental health service use among White, Chinese, South Asian and South East Asian populations living in Canada. Social Psychiatry and Psychiatric Epidemiology. 2008;43(11):866 10.1007/s00127-008-0373-6 18500481

[12] Tran TV, Manalo V, Nguyen VT. Nonlinear relationship between length of residence and depression in a community-based sample of Vietnamese Americans. International Journal of Social Psychiatry. 2007;53(1):85–94. 10.1177/0020764007075025 17333954

[13] Wu Z, Schimmele CM. The healthy migrant effect on depression: variation over time? Canadian Studies in Population. 2005;32(2):271–95.

[14] O'hara MW, Swain AM. Rates and risk of postpartum depression—a meta-analysis. International review of psychiatry. 1996;8(1):37–54.

[15] Spector AZ. Fatherhood and depression: A review of risks, effects, and clinical application. Issues in Mental Health Nursing. 2006;27(8):867–83. 10.1080/01612840600840844 16938789

[16] Collins CH, Zimmerman C, Howard LM. Refugee, asylum seeker, immigrant women and postnatal depression: rates and risk factors. Archives of women's mental health. 2011;14(1):3–11. 10.1007/s00737-010-0198-7 21153849

[17] Tang TN, Oatley K, Toner BB. Impact of life events and difficulties on the mental health of Chinese immigrant women. Journal of Immigrant and Minority Health. 2007;9(4):281–90. 10.1007/s10903-007-9042-1 17347889

[18] Beiser M. Resettling refugees and safeguarding their mental health: Lessons learned from the Canadian Refugee Resettlement Project. Transcultural psychiatry. 2009;46(4):539–83. 10.1177/1363461509351373 20028677

[19] Amato PR. Children of Divorce in the 1990s: An Update of the Amato and Keith (1991) meta-analysis. Journal of Family Psychology. 2001;15:355–70. 11584788 10.1037//0893-3200.15.3.355

[20] Ray B, Preston V. Are immigrants socially isolated? An assessment of neighbors and neighboring in Canadian cities. Journal of International Migration and Integration/Revue de l'integration et de la migration internationale. 2009;10(3):217–44.

[21] Cokley K, Hall-Clark B, Hicks D. Ethnic minority-majority status and mental health: The mediating role of perceived discrimination. Journal of Mental Health Counseling. 2011;33(3):243–63.

[22] Finch BK, Kolody B, Vega WA. Perceived discrimination and depression among Mexican-origin adults in California. Journal of Health and Social Behavior. 2000:295–313. 11011506

[23] Kim I-H, Noh S. Ethnic and gender differences in the association between discrimination and depressive symptoms among five immigrant groups. Journal of immigrant and minority health. 2014;16(6):1167–75. 10.1007/s10903-013-9969-3 24375383

[24] Telzer EH, Vazquez Garcia HA. Skin color and self-perceptions of immigrant and US-born Latinas: The moderating role of racial socialization and ethnic identity. Hispanic Journal of Behavioral Sciences. 2009;31(3):357–74.

[25] Leyendecker B, Harwood R. L., Comparini L., & Yalcinkaya A.. Socioeconomic status, ethnicity and parenting In: Luster T. O L, editor. Parenting: An ecological perspective 2nd ed: Mahwah: Lawrence Erlbaum; 2005 p. 319–42.

[26] Lorant V, Deliège D, Eaton W, Robert A, Philippot P, Ansseau M. Socioeconomic inequalities in depression: a meta-analysis. American Journal of Epidemiology. 2003;157(2):98–112. 12522017 10.1093/aje/kwf182

[27] Myers HF. Ethnicity-and socio-economic status-related stresses in context: an integrative review and conceptual model. Journal of behavioral medicine. 2009;32(1):9–19. 10.1007/s10865-008-9181-4 18989769

[28] Hansson EK, Tuck A, Lurie S, McKenzie K. Rates of mental illness and suicidality in immigrant, refugee, ethnocultural, and racialized groups in Canada: a review of the literature. The Canadian Journal of Psychiatry. 2012;57(2):111–21. 10.1177/070674371205700208 22340151

[29] Walks R, Bourne LS. Ghettos in Canada's cities? Racial segregation, ethnic enclaves and poverty concentration in Canadian urban areas. The Canadian Geographer/Le Géographe canadien. 2006;50(3):273–97.

[30] Schellenberg G. Immigrants in Canada's census metropolitan areas: Statistics Canada, Business and Labour Market Analysis Division Ottawa; 2004.

[31] Chen C, Smith P, Mustard C. The prevalence of over-qualification and its association with health status among occupationally active new immigrants to Canada. Ethnicity & health. 2010;15(6):601–19. 20859815 10.1080/13557858.2010.502591

[32] Statistics Canada. Microdata User Guide: Longitudinal Survey of Immigrants to Canada, Wave 3. Ottawa, Canada: 2007 http://www23.statcan.gc.ca/imdb-bmdi/document/4422_D1_T1_V3-eng.pdf

[33] Statistics Act c.S-19, Government of Canada(1985). http://laws-lois.justice.gc.ca/eng/acts/S-19/FullText.html

[34] Fitzmaurice GM, Laird NM, Ware JH. Applied longitudinal analysis: John Wiley & Sons; 2012.

[35] Hedeker D, Gibbons RD. Longitudinal data analysis. Hoboken, NJ: John Wiley & Sons; 2006.

[36] Singer JD, Willett JB. Applied longitudinal data analysis: Modeling change and event occurrence. London: Oxford University Press; 2003.

[37] Simon RW. Revisiting the Relationships among Gender, Marital Status, and Mental Health 1. American journal of sociology. 2002;107(4):1065–96. 10.1086/339225 12227382

[38] Kandel DB, Davies M, Raveis VH. The stressfulness of daily social roles for women: Marital, occupational and household roles. Journal of Health and Social Behavior. 1985:64–78. 3998436

[39] Pearlin LI, Johnson JS. Marital status, life-strains and depression. American sociological review. 1977:704–15. 931191

[40] Avison WR, Davies L. Family structure, gender, and health in the context of the life course. The Journals of Gerontology Series B: Psychological Sciences and Social Sciences. 2005;60(Special Issue 2):S113–S6. 10.1093/geronb/60.special_issue_2.s113 16251581

[41] Hyman I, Guruge S, Mason R. The impact of migration on marital relationships: A study of Ethiopian immigrants in Toronto. Journal of Comparative Family Studies. 2008:149–63.

[42] Kossoudji SA. English language ability and the labor market opportunities of Hispanic and East Asian immigrant men. Journal of Labor Economics. 1988;6(2):205–28.

[43] Takeuchi DT, Zane N, Hong S, Chae DH, Gong F, Gee GC, et al Immigration-related factors and mental disorders among Asian Americans. American Journal of Public Health. 2007;97(1):84–90. 10.2105/AJPH.2006.088401 17138908 PMC1716230

[44] Noh S, Kaspar V. Perceived discrimination and depression: Moderating effects of coping, acculturation, and ethnic support. American journal of public health. 2003;93(2):232–8. 12554575 10.2105/ajph.93.2.232 PMC1447722

[45] Weissman MM, Bland RC, Canino GJ, Faravelli C, Greenwald S, Hwu H-G, et al Cross-national epidemiology of major depression and bipolar disorder. JAMA. 1996;276(4):293–9. 8656541

[46] Bradley RH, Corwyn RF. Socioeconomic Status and Child Development. Annu Rev Psychol. 2002:235–309. 10.1146/annurev.psych.53.100901.135233 11752490

[47] Almeida J, Subramanian S, Kawachi I, Molnar BE. Is blood thicker than water? Social support, depression and the modifying role of ethnicity/nativity status. Journal of epidemiology and community health. 2011;65(1):51–6. 10.1136/jech.2009.092213 19910646

[48] Lefèvre T, Singh-Manoux A, Stringhini S, Dugravot A, Lemogne C, Consoli SM, et al Usefulness of a single-item measure of depression to predict mortality: the GAZEL prospective cohort study. The European Journal of Public Health. 2012;22(5):643–7. 10.1093/eurpub/ckr103 21840893 PMC3457003

[49] Karasz A. Cultural differences in conceptual models of depression. Social science & medicine. 2005;60(7):1625–35. 15652693 10.1016/j.socscimed.2004.08.011

[50] Sanchez F, Gaw A. Mental health care of Filipino Americans. Psychiatric Services. 2007;58(6):810–5. 10.1176/ps.2007.58.6.810 17535941

