# Supplementary material for: Emotional problems among recent immigrants and parenting status: Findings from a national longitudinal study of immigrants in Canada
Source: PLoS One. 2017 Apr 4;12(4):e0175023. doi: 10.1371/journal.pone.0175023 (PMC5380348; doi:10.1371/journal.pone.0175023)
Supplement: S1 Appendix — (PDF) [file pone.0175023.s001.pdf]

Project ID number: 11-SSH-UTO-2879 *Revised: August 18, 2011*
Title: Mental Health Among New Canadian Immigrants: Examining the Effects of Family Structure and Ethnicity using a National Longitudinal Database

MICRODATA RESEARCH CONTRACT

BETWEEN:

HER MAJESTY THE QUEEN IN RIGHT OF CANADA, represented by the Minister of Industry, designated as the Minister for purposes of the Statistics Act, (hereinafter referred to as "Statistics Canada"),

AND:

Aarti Kumar, University of Toronto
(hereinafter referred to as the "Principal Investigator"),

and,

Olesya Falenchuk, University of Toronto
Dillon Browne, University of Toronto
(hereinafter referred to as the "Co-investigators"),

WHEREAS Statistics Canada requires the services of the Principal Investigator to undertake statistical research and analysis on *Longitudinal Survey of Immigrants to Canada (LSIC)* to fulfill its mandate under the *Statistics Act*;

AND WHEREAS to perform these services and to have access to confidential information, the Principal Investigator and the Co-investigators must become "Deemed Employees" of Statistics Canada and are required to take the Oath of Secrecy;

AND WHEREAS Statistics Canada wishes to make clear the terms and conditions under which access to the microdata will be granted;

NOW THEREFORE the Parties agree as follows:

SERVICE PROVIDED BY PRINCIPAL INVESTIGATOR

1. (1) The Principal Investigator will carry out the research project set out in Appendix "A" and provide the report described under "Proposed Output".

   (2) It is understood that this is a contract for the performance of a service and the Principal Investigator and Co-investigators are engaged for the sole purpose of

providing that service.

CONDITIONS OF ACCESS TO THE MICRODATA

2. The Principal Investigator and the Co-investigators must undergo an enhanced reliability check satisfactory to Statistics Canada and take the oath/affirmation of secrecy, pursuant to Subsection 6(1) of the Statistics Act, in order to obtain access to the non identifiable microdata file required to perform the analysis pursuant to this contract.

3. (1) Access to the non identifiable microdata file (no names, addresses or identifying numbers) and associated documentation shall be provided on Statistics Canada premises, which includes Headquarters and the Statistics Canada Regional Offices during normal hours of operation, Monday to Friday, and the Research Data Centres.

(2) The Principal Investigator and Co-Investigators will be provided with the necessary computing facilities, software and documentation as is reasonably necessary to complete the research and analysis pursuant to this contract.

DEPARTMENTAL REPRESENTATIVE

4. The *Manager of the Microdata Access Division* is the designated Statistics Canada representative responsible for the administration of this contract.

LIMITATIONS ON USES OF THE MICRODATA FILE AND PROPOSED OUTPUT

5. (1) No person engaged in the course of carrying out this contract may use any of the information gained by accessing the confidential data for any other purpose except that which was agreed upon in this contract.

(2) Access to the microdata file is being provided for the statistical and research purpose outlined in the proposal attached as Appendix A and the microdata file shall not be used for any other purposes without the prior written consent of Statistics Canada.

(3) The Principal Investigator and the Co-investigators shall not disclose any of the information from the individual records obtained or produced pursuant to this contract to anyone other than current Statistics Canada employees involved in the review or evaluation of any aspect of the research project.

(4) The Principal Investigator and the Co-investigators shall ensure that no attempts are made to link the microdata file to any other files in order to relate the particulars to any identifiable individual person, business or organization.

(5) The Proposed Output must meet the requirements of both peer and institutional review prior to being released by Statistics Canada, for example, in one of its publications or in a research paper.

(6) Thereafter, the Principal Investigator may, subject to subsection 6(5), carry out a secondary analysis, but such analysis shall be based solely on the approved "Proposed Output" produced pursuant to this contract and be related to the analytical work undertaken to produce the "Proposed Output".

(7) The Principal Investigator agrees to work with Statistics Canada in trying to meet the requirements of peer and institutional review required for the publication or research paper.

(8) In the event that Statistics Canada decides not to publish the "Proposed Output", Statistics Canada will give the Principal Investigator written notice to this effect within thirty days of having made the final decision.

(9) Subject to subsections 6(5) and 10(2), in the event Statistics Canada notifies the Principal Investigator in writing that the proposed output will not be published, the Principal Investigator may:

(a) Publish the "Proposed Output" elsewhere, and/or

(b) Use the "Proposed Output" for purposes of the attainment of an educational degree.

OWNERSHIP

6. (1) The microdata file and related documentation shall at all times be and remain the sole and exclusive property of Statistics Canada, it being mutually agreed that this contract pertains to the use of the microdata file and related documentation to produce a "Proposed Output" for Statistics Canada and that nothing contained herein shall be deemed to convey any title or ownership interest in the microdata file or the related documentation to the Principal Investigator or the Co-investigators. The computer equipment provided for use by the Principal Investigator and the Co-investigators must never be removed from the premises of Statistics Canada or the Research Data Centre and remains the sole and exclusive property of the access facility.

(2) Statistics Canada reserves the right to publish in whole or in part, to publish an amended version or not to publish at all, as Statistics Canada deems appropriate, the "Proposed Output" produced by the Principal Investigator pursuant to this contract.

(3) Copyright in the "Proposed Output" produced by the Principal Investigator pursuant to this contract shall vest in Her Majesty the Queen in Right of Canada. The Principal Investigator shall provide to Statistics Canada at the completion of the contract or at such other time as Statistics Canada may require a written permanent waiver of Moral rights from every author who contributed to the aforementioned material.

(4) Statistics Canada (Her Majesty the Queen in Right of Canada) hereby grants to the Principal Investigator a non-exclusive license to use, reproduce, publish and distribute the "Proposed Output" for any purpose, including, without limitation, research, teaching and publication in any medium. Copyright in any subsequent work produced by the Principal Investigator using the "Proposed Output" shall vest in the Principal Investigator.

(5) Releases of the "Proposed Output" may be considered by Statistics Canada in consultation with the Principal Investigator.

(6) In publishing the "Proposed Output" elsewhere, using the "Proposed Output" in the attainment of an educational degree or carrying out any secondary analysis, any reports, documents, or materials which are subsequently prepared by the Principal Investigator which use, incorporate or are in any way based on any material produced under this agreement, shall prominently display the following notice:

"The research and analysis are based on data from Statistics Canada and the opinions expressed do not represent the views of Statistics Canada."

CONFLICT OF INTEREST

	Your initials
7. (1) All persons engaged in the course of carrying out this contract shall conduct themselves in accordance with the principles and spirit of the *Values and Ethics Code for Deemed Employees*.	
Insert below names of all Deemed Employees associated with this Contract:	
Aarti Kumar	AK
Olesya Falenchuk	OF
Dillon Browne	DB

(2) Should a conflict exist prior to the commencement of this contract or be acquired or develop during the life of this contract, the person with the conflict engaged in carrying out this contract shall be responsible for discussing the conflict with the Manager of the Microdata Access Division and, should it be determined that a conflict exists, for completing the Confidential Report as required by the *Values and Ethics Code for Deemed Employees*

(3) No person engaged in the course of carrying out this contract may use any of the information gained by accessing the confidential data for any other purpose except that which was agreed upon in this contract.

(4) Notwithstanding subsection 7(3), it is understood that the principles of the *Values and Ethics Code for Deemed Employees* were not meant to prohibit the persons engaged in this contract from accomplishing any secondary analysis as permitted by the contract.

SECURITY REQUIREMENTS

8. (1) Any material to be removed from the Statistics Canada premises by the Principal Investigator or Co-investigators must first be screened by Statistics Canada to ensure that there is no risk of disclosure of confidential information or information that may lead to the identification of an individual person, business or organization. It is the responsibility of the Principal Investigator or Co-investigators to take all precautions to avoid disclosure of confidential information or information that may lead to the identification of an individual respondent. The Principal Investigator or Co-investigators may remove summary files, tabulations and analytical output under the terms of this subsection.

(2) The Principal Investigator and the Co-investigators shall not remove any of the original data set or copies of subsets of the microdata file or any confidential sensitive statistical information provided pursuant to this contract from the premises of Statistics Canada.

(3) The Principal Investigator and the Co-investigators shall be provided with copies of all relevant Statistics Canada policies related to confidentiality, privacy and security and the standard operating procedures of the appropriate access facility and shall acknowledge in writing their compliance with all of the policies and operating procedures.

TERM

9. This contract comes into force when signed by both parties and shall continue in force until *31 August 2012* unless revoked or terminated at an earlier date.

TERMINATION

10. (1) Where the Principal Investigator is in default in carrying out any of its obligations under this Contract, Statistics Canada may, upon giving written notice to the Principal Investigator, terminate the Contract immediately.

(2) The Contract may, by providing 30 days written notice, be terminated by mutual written consent between the Principal Investigator and Statistics Canada.

(3) Any notice to be given to Statistics Canada or the Principal Investigator shall be sent by registered mail to:

Heather Dryburgh, Ph.D.<br>Manager, Microdata Access Division<br>Statistics Canada<br>150 Tunney's Pasture Driveway<br>Ottawa, Ontario K1A 0T6	*Aarti Kumar*<br>*Principal Investigator*<br>*280 St. George Street, Apt. 1206*<br>*Toronto, Ontario*<br>*M5R2P7*

(4) Any notice shall be deemed to be effective on the day it is received at the address set out above.

PENALTIES

11. (1) As a Deemed Employee of Statistics Canada, the Principal Investigator and the Co-investigators are subject to all the applicable penalties provided for in the *Statistics Act* for contravention of any of the confidentiality provisions and is liable on summary conviction to any of the applicable fines or imprisonment terms.

(2) Subsection 11(1) survives indefinitely the completion of this contract or the termination of this Agreement pursuant to subsections 10(1) or 10(2).

AMENDMENT

12. No amendment to this contract shall be valid unless it is reduced to writing and signed by the Parties hereto.

CONSIDERATION

13. The Parties agree that consideration for this agreement shall be the mutual promises and covenants of the Parties included in this contract.

ENTIRE AGREEMENT

14. This contract constitutes the entire agreement between the Parties listed below and Statistics Canada with respect to the subject matter described herein and supersedes all previous negotiations, communications and other agreements on the same issue with Statistics Canada unless they are specifically incorporated by reference in this contract.

IN WITNESS WHEREOF, this Agreement has been executed in duplicate on behalf of Statistics Canada and the Principal Investigator by:

FOR STATISTICS CANADA:

Witness	Aug 23/11<br>Date	Manager of the Microdata Access Division (as described in paragraph 4 above)

FOR THE PRINCIPAL INVESTIGATOR:

Witness	Aug 23/11<br>Date	*Aarti Kumar*, Principal Investigator
Witness	Aug 23/11<br>Date	*Olesya Falenchuk*, Co-investigator
Witness	Aug 23/11<br>Date	*Dillon Browne*, Co-investigator

Appendix "A"

Mental Health Among New Canadian Immigrants: Examining the Effects of Family Structure and Ethnicity using a National Longitudinal Database

Submitted By: Aarti Kumar
280 St. George Street, Apt. 1206
Toronto, Ontario M5R2P7
905- 336-3252

Proposal

See attached proposal.

Data Requirements

LSIC

Proposed Output

- Peer-reviewed journal article: A journal article authored by the Principal Investigator for a peer-reviewed journal.

Completion Date

31 August 2012

Research Location

Toronto RDC

Source of Funding

N/A
