# Supplementary material for: Emotional problems among recent immigrants and parenting status: Findings from a national longitudinal study of immigrants in Canada
Source: PLoS One. 2017 Apr 4;12(4):e0175023. doi: 10.1371/journal.pone.0175023 (PMC5380348; doi:10.1371/journal.pone.0175023)
Supplement: S2 Appendix — (DOCX) [file pone.0175023.s002.docx]

**S2 Appendix**

Please note that a comprehensive description of the LSIC is available online: <http://www23.statcan.gc.ca/imdb-bmdi/pub/4422-eng.htm>

**Supplementary Description of Income Measurement**

At 6 months post-immigration, respondents were asked to provide their total income from all sources over the past 6 months, while at 2 years and 4 years post-immigration, they were asked to provide this over the past 12 months. To establish the same metric across time an average monthly income variable was computed. Due to an extreme positive skew (i.e. some economic immigrants earning very high incomes), a “low income” variable was computed based on the sample (n.b. a log transformation did not resolve the skewness problem). Dichotomous variables at less than vs. equal to or greater than the 15^th^, 20^th^ and 25^th^ percentiles were constructed and tested in models. Results did not vary across different percentile cut-offs. As a result the 20^th^ percentile was used as the low-income cut-off. The 20^th^ percentile monthly income at time 1, 2, and 3 was $ 466.67, $ 1490.00 and $ 2018.27, respectively. This corresponds to 20^th^ percentile annual incomes of $ 5600.04, $17,800.00 and $ 24,219.24 across times, respectively. All income values are in Canadian Dollars. Although Statistics Canada provides Low Income Cut-Off’s (LICO) for the nation, the distribution of income in immigrants is significantly lower than the general population, with a large number of immigrants falling well below the LICO cut-offs. As a result, the LICOs were not applied, and a specific low-income cut-off was created using the existing LSIC sample. Also, income was specified as a time varying predictor due to the large increases in income over the first years in a host country.

**Supplementary Description of Education Measurement**

Number of years of formal full-time education was collected at time 1. Respondents were asked, “In total, how many years of full-time education have you completed, both inside and outside Canada, excluding kindergarten?” Updates on this variable were not collected in subsequent waves of data collection.

**Supplementary Description of Immigration Category Measurement**

Respondents were asked to which immigration category they belonged based on criteria from the Field Operated Support System (FOSS). The original immigration categories were collapsed into the following five immigrant groups; (1) Family class (Family class – Spouses and Fiancés (principal applicants), Family class – Parents and Grandparents (principal applicants and spouses) and Family class – Other), (2) Business Immigrants (Business Immigrants (principal applicants) and Business Immigrants (spouses and dependents)), (3) Refugees (Government Sponsored Refugee, Privately Sponsored Refugees and Other Refugees Abroad), (4) Skilled Workers (Skilled Workers (principal applicants) With Relatives in Canada, Skilled Workers (principal applicants) Without Relatives in Canada, and Skilled Workers (spouses and dependents) Without Relatives in Canada)), and (5) Other Immigrants (Provincial Nominees (principal applicants), Provincial Nominees (spouses and dependents), Other Immigrants Abroad). A series of dummy codes were created for the above immigrant categories, with Skilled Workers as the reference category. Any longitudinal respondent that did not fit into these categories were excluded from the analysis (Valid skip, Don’t Know, Refused and Not Stated).
**Supplementary Description of Ethnicity Measurement**

Respondents were asked which ethnic group they identified with from the following categories established by Statistics Canada, “White” (1), “Chinese” (2), “South Asian (e.g., East Indian, Pakistani, Sri Lankan, etc.)” (3), “Black” (4), “Filipino” (5), “Latin American” (6), “Southeast Asian (e.g., Cambodian, Indonesian, Laotian, Vietnamese, etc.)” (7), “Arab” (8), “West Asian (e.g., Afghan, Iranian, etc.)” (9), “Japanese” (10), “Korean” (11), “Visible Minority Not Identified Elsewhere” (12), “Multiple Visible Minorities” (13), “Don’t know” (97), “Refused” (98). The original ethnic groups were collapsed to the following broader ethnic groups; White, South Asian, East Asian (Chinese, Japanese, Southeast Asian and Korean), Black, Arab/West Asian, Latin American, and Filipino. Ethnic groups were dummy-coded with “White Immigrants” being the reference category. Any longitudinal respondent that responded as either *Multiple Visible Minorities,* *don’t know* or *refusal*, were excluded from the analysis (N=86).

**Supplementary Description of Parenting Measurement**

Preliminary analyses were carried out to construct properly differentiated variables to represent the construct of parenting status. Several variables were relevant. Marital status captured whether the respondent was married, single or divorced. The parent variable captured whether the respondent had children living in the household. In terms of marital status, our goal was to isolate the effects of being a parent, while appropriately accounting for different types of marital transition, which covary with being a parent. Both ‘marital status’ and ‘parent’ can vary over time. The decision was made to treat predictors as time invariant. Combinations of marital status, time, and parenting status resulted in 16 combinations: (1) Married at all time points and children all time points, (2) Married at all time points and never had children; (3) Married at all time points and no children to children at some time point (4) Married at all time points and children to no children at some time point ; (5) Single at all time points and children at all time points; (6) Single at all time points and never had children; (7) Single at all time points and no children to children at some time point; (8) Single at all time points and children to no children at some time point; (9) Divorced at any time point and children at all time points; (10) Divorced at any time point and never had children; (11) Divorced at any time point and no children to children at some time point; (12) Divorced at any time point and children to no children at some time point; (13) Single to married and children at all time points; (14) Single to married and never had children; (15) Single to married and no children to children at some time point; (16) Single to married and children to no children at some time point.

A total of 5 groups had cell sizes between 0 – 10 and were collapsed with groups that were theoretically similar. The remaining 11 groups were examined to create homogenous categories. When the outcome variable was not statistically different and fit together based on theoretical principles, they were collapsed into one group. For example, respondents who reported being married at all time points and single to married were not statistically different and collapsed into one group. In addition, models testing differences between respondents who had children at all time points, no children to children, and children to no children were not statistically significant and collapsed together. These groupings were made because the reasons for changes in number of children over time were unknown (reunification, new born baby, departure from home based on age, loss etc.) and could not be grouped based on theoretical understanding.

The final groupings are as follows: Two parent, Lone parent, Divorced non-parent and Non-parent. *Two parent* was defined as caring for one or more children equal to or less than 18 years of age at any time point and identifying themselves as either “married” or “common-law” at all time points or “single” to “married” at any time point. *Lone parent* was defined as a parent caring for one or more children equal to or less than 18 years of age at any time point and identifying themselves as divorced, separated or widowed at either time 1, 2 or 3. *Divorced non-parent* was defined as the person not being a parent at all time points and being divorced, separated or widowed at either time 1, 2 or 3. *Non-Parent* (the reference category) refers to respondents who are not caring for children equal to or less than 18 years of age at all cycles and are either partnered or never married. All parenting status variables were mutually exclusive. Sensitivity analyses were conducted to examine associations with the outcome for all groups within a category to ensure consistency in the reported associations.

**Supplemental Material on Sensitivity Analyses**

Patterns of self-reported emotional problems were modeled in several different ways. That is, sensitivity analyses were conducted to see if model assumptions impacted substantive results. Multilevel Mixed Effects Logistic Regression was conducted first, which is the standard subject-specific approach for modeling outcomes that are both repeated measures and dichotomous. Preliminary analyses demonstrated significant within-subject dependencies in the response variables, thereby requiring a model that accounts for this non-independence. Similar to regular Logistic Regression, a Binomial distribution was assumed and the Logit Link was employed. A random intercept can be included which allows the intercept to vary across subjects. When this term is significant, it indicates that there is significant between-subject variability in the tendency to report emotional problems that is not being accounted for by variables in the model. The above multilevel approach does not permit the employment of survey weights, as the weights are assigned at the person level and cannot represent the within-person portion of the model. Thus, a population-averaged approach (i.e. a marginal model) was also tested using Generalized Estimating Equations, with and without survey weights. Results were substantively identical. Due to reasons cited in the missing data section, parsimony and model invariance, the least restrictive model is reported below (i.e. Multilevel Mixed Effects Logistic Regression).

**Supplemental Material on Missing Data**

Missing data is described in accordance with recommendations outlined by Graham [1]. However, statistical techniques to account for missingness (e.g. Multiple Imputation) were not appropriate due to the categorical nature of the data. That is, missing data imputation and estimation techniques are much less developed for categorical data (both outcomes and predictors) compared to continuous data [2]. Previous studies have suggested that Multiple Imputation can produce biased estimates when employed in conjunction with categorical data. Moreover, population survey weights are inappropriate to use when 100% of the sample is not included in the analysis [3]. That is, the sample weight for an individual participant is computed based on their representativeness of the population relative to all other persons in the data set. Given these limitations, the unweighted and non-imputed model output was deemed to be the most reliable. Nevertheless, models were run in several different ways (see above) with no observable difference. The unweighted and non-imputed results are reported, but substantive results did not change as a function of missing data treatment. Missing data analyses did not suggest any systematic patterns of missingness based on risk (i.e., there were no differences between individuals with missing data versus those with complete data on EP, income or visible minority status).

**Supplementary Description of Construct Validity in Emotional Problem Measurement**

It is important to consider construct validity in the measurement of emotional problems. In psychiatric epidemiology, emotional problems are typically evaluated through self-report metrics. Given the substantial costs associated with mental health screening, the predictive and convergent validity of short screens is of particular interest. Often, these screens include single item measurement. For example, Lefèvre and colleagues [4] demonstrated excellent predictive validity of a single item for depression (“I felt depressed”) in the prediction of all-cause mortality among a large French cohort (N≈14,000). Similar findings have been documented in the study of psychosocial stress [5] and generic quality of life [6]. Based on simulation studies, single item responses are sufficient when large samples are used and the construct is measured with high internal consistency on multi-item scales [7]. The present investigation examined emotional problems using a single item (see Measures section). The validity of this approach is supported by a large sample and previous demonstrations of the coherence of the emotional problems construct [8].

Given the variability in measuring emotional well-being, it is worth comparing and contrasting the current study results with similar investigations that employed alternative measurement strategies. Our findings are consistent with Chung and colleagues [9] findings of increasing depression and anxiety (collectively, psychological distress) across the post-migration years. In this study, separate multi-item anxiety and depression composites were used and findings were similar across dimensions. Similarly, findings connecting parenting status and emotional problems are consistent with studies that have used more comprehensive screens of psychological distress. For example, our findings replicate McKenzie & Carter’s [10] finding linking parenthood and psychological distress, measured by the widely used the Short-Form 36 (positive and negative emotion items) [11] and the Kessler-10 Distress Scale (non-specific psychological distress) [8]. These comparisons suggest that our dependent measure accesses respondents’ subjective experience of psychological malaise.

**References**

1. Graham JW. Missing data analysis: Making it work in the real world. Annual Review of Psychology.

2009;60:549-76. doi: [10.1146/annurev.psych.58.110405.085530](https://doi.org/10.1146/annurev.psych.58.110405.085530)

2. McKnight PE, McKnight KM, Sidani S, Figueredo AJ. Missing data: A gentle introduction: Guilford

Press; 2007.

3. Lee ES, Forthofer RN. Analyzing complex survey data: Sage Publications; 2005.

4. Lefèvre T, Singh-Manoux A, Stringhini S, Dugravot A, Lemogne C, Consoli SM, et al. Usefulness of a

single-item measure of depression to predict mortality: the GAZEL prospective cohort study. The

European Journal of Public Health. 2012;22(5):643-7. doi: [10.1093/eurpub/ckr103](https://doi.org/10.1093/eurpub/ckr103)

5. Elo A-L, Leppänen A, Jahkola A. Validity of a single-item measure of stress symptoms. Scandinavian

journal of work, environment & health. 2003:444-51.

6. De Boer A, Van Lanschot J, Stalmeier P, Van Sandick J, Hulscher J, De Haes J, et al. Is a single-item

visual analogue scale as valid, reliable and responsive as multi-item scales in measuring quality

of life? Quality of Life Research. 2004;13(2):311-20. doi: [10.1023/b:qure.0000018499.64574.1f](https://doi.org/10.1023/b:qure.0000018499.64574.1f)

7. Diamantopoulos A, Sarstedt M, Fuchs C, Wilczynski P, Kaiser S. Guidelines for choosing between

multi-item and single-item scales for construct measurement: a predictive validity perspective.

Journal of the Academy of Marketing Science. 2012;40(3):434-49. doi: [10.1007/s11747-011-0300-3](https://doi.org/10.1007/s11747-011-0300-3)

8. Kessler RC, Andrews G, Colpe LJ, Hiripi E, Mroczek DK, Normand S-L, et al. Short screening scales

to monitor population prevalences and trends in non-specific psychological distress.

Psychological medicine. 2002;32(06):959-76. doi: [10.1017/s0033291702006074](https://doi.org/10.1017/s0033291702006074)

9. Chung RCY, Kagawa-Singer, M. *Predictors of psychological distress among Southeast Asian*

*refugees.* Social science & medicine. 1993;36(5):631-639. doi: 10.1016/0277-

9536(93)90060-h

10. Mckenzie SK, Carter, K. *Does transition into parenthood lead to changes in mental health?*

*Findings from three waves of a population based panel study.* Journal of epidemiology

and community health. 2012;67(4):339-345. doi: [10.1136/jech-2012-201765](https://doi.org/10.1136/jech-2012-201765)

11. Brazier JE, Harper R, Jones NM, O’Cathain A, Thomas KJ, Usherwood T, et al. *Validating*

*the SF-36 health survey questionnaire: new outcome measure for primary care.* BMJ.

1992; 305(6846):160-164. doi: [10.1136/bmj.305.6846.160](https://doi.org/10.1136/bmj.305.6846.160)
